# Prolonged Hospitalization in a Pediatric Patient With Multisystem Inflammatory Syndrome (MIS-C) and Human Rhino-Enterovirus Infection: A Case Report

**DOI:** 10.7759/cureus.29380

**Published:** 2022-09-20

**Authors:** Ashley Lee, Ivana Stojkic, Ekemini A Ogbu

**Affiliations:** 1 Allergy and Immunology, Icahn School of Medicine at Mount Sinai, New York, USA; 2 Pediatrics, Nationwide Children’s Hospital, Columbus, USA; 3 Rheumatology, Cincinnati Children's Hospital Medical Center, Cincinnati, USA; 4 Pediatric Rheumatology, University of Cincinnati College of Medicine, Cincinnati, USA; 5 Pediatrics, Johns Hopkins University School of Medicine, Baltimore, USA

**Keywords:** rhinovirus, enterovirus, sars-cov-2, covid-19, mis-c

## Abstract

Multisystem inflammatory syndrome in children (MIS-C) is a serious sequela of acute SARS-CoV-2 infection. It is unclear whether the co-occurrence of other viral respiratory illnesses, such as the human rhino-enterovirus (HRV/ENT), prolongs hospitalization or affects the clinical phenotype of patients with MIS-C. We report the hospital course of a three-year-old with MIS-C and HRV/ENT infection, who tested positive for HRV/ENT infection a few days prior to re-presenting for six days of fever, one day of emesis, bilateral conjunctivitis, and shortness of breath, all consistent with MIS-C. Due to worsening hypotension, he was admitted to a pediatric intensive care unit (ICU) at a tertiary center, where he received vasoactive support, intravenous immunoglobulin, and high-dose intravenous steroids. Because of his worsening respiratory status, he was also started on anakinra with resultant gradual improvement. He was hospitalized for a total of 15 days. Concurrence of other viral infections may prolong hospitalization for patients with MIS-C.

## Introduction

Multisystem inflammatory syndrome in children (MIS-C) is a post-infectious inflammatory syndrome with a spectrum of presentations, including fever, mucocutaneous, gastrointestinal, cardiorespiratory, and/or neurological symptoms [1]. MIS-C typically occurs two to four weeks following an acute SARS‐CoV‐2 infection. As of May 2022, over 4,000 cases of MIS-C had been reported in the United States [1]. Several studies investigating the clinical outcomes of pediatric patients with MIS-C in the United States suggest that the mean or median duration of hospitalization for MIS-C is seven days [2-4]. Our patient, who presented with severe multisystem disease including neurologic involvement, was admitted for a total of 15 days. It is possible that his hospital course and refractory respiratory features were compounded by his HRV/ENT infection. Furthermore, the impact of viral co-infection on MIS-C clinical phenotype is yet to be fully elucidated.

## Case presentation

**Figure 1:**
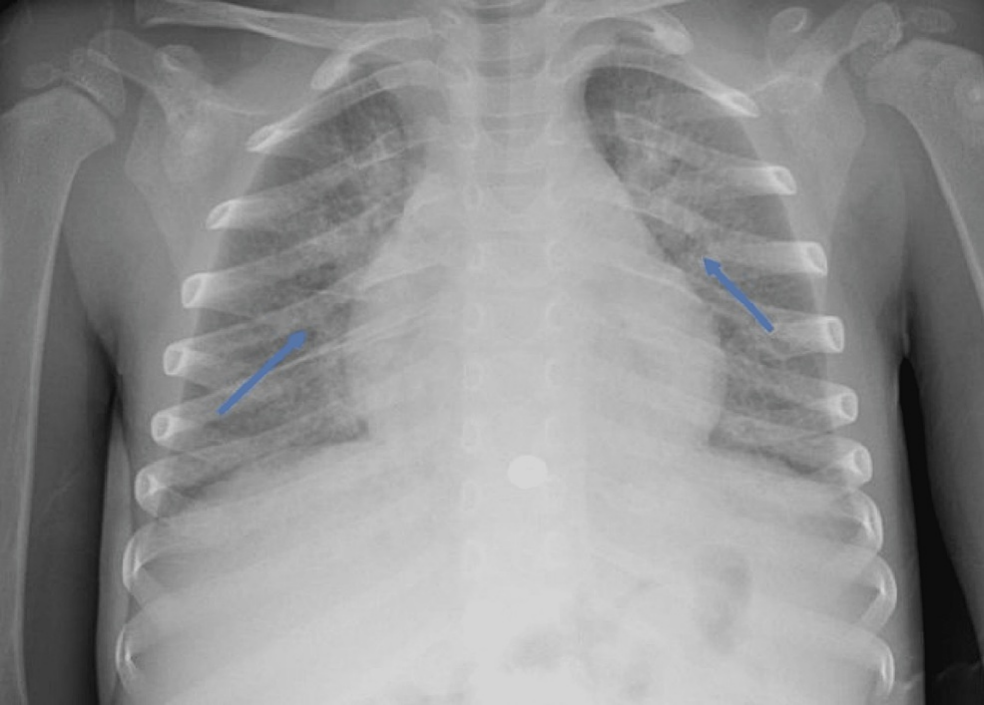
Chest radiograph of a three-year-old patient with Multisystem Inflammatory Syndrome in Children showing diffuse hazy, peri-hilar densities and peri-bronchial cuffing (blue arrows)

We report the case of a three-year-old male who presented to an outside emergency room (ER) with six days of fever and one day of bilateral conjunctivitis, emesis, and shortness of breath. He was initially evaluated in the same ER for fever three days prior. At that time, he had tested positive for rhino-enterovirus (HRV/ENT), but negative for severe acute respiratory syndrome coronavirus-2 (SARS‐CoV‐2) by nasopharyngeal PCR. Upon re-presenting to the ER, he was febrile to 39°C and tachypneic to 60 breaths/min. His oxygen saturation was 98% on room air. He had bilateral conjunctival injection. His laboratory data showed hyponatremia with sodium of 126 mmol/L (range 135-148). C-reactive protein was elevated at greater than 190 mg/L (range 0.00-3.00). His SARS‐CoV‐2 nasopharyngeal PCR was positive. His chest radiograph showed hazy peri-hilar densities and peri-bronchial cuffing (Figure 1). He had a brief, self-resolved seizure with unilateral eye deviation and refractory hypotension prompting transfer to a tertiary pediatric intensive care unit (ICU).

**Figure 2:**
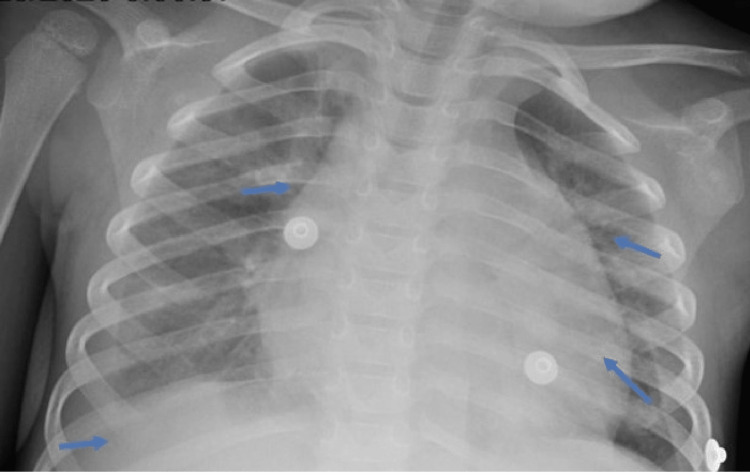
Subsequent chest radiograph of the same three-year-old patient with Multisystem Inflammatory Syndrome in Children showing a mildly enlarged cardiothymic silhouette, bilateral pulmonary opacities and trace bilateral pleural effusions (blue arrows)

In the pediatric ICU, he was started on 2 liters of oxygen via nasal cannula, intravenous fluids, vasopressors, and empiric antibiotics. Additional workup revealed anemia with thrombocytopenia, slightly improved hyponatremia since admission, elevated ferritin, triglyceride, troponin, d-dimer, and urine protein to creatinine ratio (Table 1). His SARS‐CoV‐2 immunoglobulin A and G antibodies were positive. His chest radiograph showed diffuse pulmonary opacities with trace bilateral pleural effusions (Figure 2). His ECHO showed normal biventricular functional and coronary arteries. He received intravenous immunoglobulin (IVIG) at 2 g/kg on admission, and was placed on high-dose intravenous (IV) methylprednisolone, aspirin, and enoxaparin. He defervesced after IVIG infusion with some improvement of his inflammatory markers and was weaned off vasopressors and supplemental oxygen by day 4 of hospitalization. However, he shortly developed increased work of breathing, tachypnea, and desaturations requiring re-initiation of supplemental oxygen. His chest X-ray showed worsening right-sided pleural effusion, and his ECHO showed a small pericardial effusion but normal function and coronaries. He was started on anakinra on day 5 of hospitalization and completed a six-day course. His inflammatory markers continued to improve, and he was weaned to room air on day 10. He was discharged on day 15 of hospitalization on aspirin and an oral prednisolone taper. He is followed longitudinally by pediatric cardiology and rheumatology.

**Table 1:** Laboratory results of our patient

Laboratory test	Laboratory values in the intensive care unit	Reference range
Sodium	130	135-148 mmol/L
Hemoglobin	9.4	11.6-13.6 g/dL
Platelet	139	150-350 K/mm^3^
Albumin	2.9	3.5-5.3 g/dL
Ferritin	591	30-400 ng/mL
Triglyceride	305	0-150 mg/dL
Pro-BNP	1766	0-125 pg/mL
Troponin	0.09	<0.04 ng/mL
d-dimer	1.16	0-0.49 mg/L
Urine protein to creatinine ratio	2.55	0.00-0.19 mg/mg

## Discussion

Our patient presented with severe MIS-C, evidenced by his clinical features and laboratory findings. He also had a concurrent HRV/ENT infection. The reported rate of other respiratory viral coinfection in pediatric patients with SARS‐CoV‐2 has varied from 3-12% [5]. While bacterial co-infection is generally accepted to lead to a worse prognosis in acute SARS‐CoV‐2 infection, the impact of viral coinfections in MIS-C remains understudied [5]. A recent study showed that respiratory viral coinfections may contribute to the increased oxygen requirement in MIS-C [6]. However, respiratory syncytial virus infection (RSV) was most common in that population [6]. Prior to the SARS‐CoV‐2 pandemic, rhino/enterovirus was responsible for one-half to two-thirds of common colds. HRV/ENT has also been associated with a more severe hospitalization course than RSV and influenza A/B infections [7]. While there are studies looking at the epidemiology of respiratory viral coinfections in children, and case reports of co-infection with COVID-19 and mycoplasma or human metapneumoviruses in children [8], there is limited data on whether HRV/ENT in MIS-C specifically leads to adverse outcomes, including prolonged hospitalization.

Treatment of hospitalized patients with MIS-C often involves IVIG and steroids. There is some evidence that initiation of combination therapy with IVIG and steroids, compared to IVIG alone, is associated with a lower rate of treatment failure, escalation to biologic therapy, and decreased length of stay [9]. Anakinra (an IL-1 receptor antagonist), has been used for off-label treatment of severe MIS-C based on clinical expertise. Currently, there are limited pediatric studies on the impact of subcutaneous or intravenous anakinra on the duration of hospitalization in MIS-C. Our case highlights that concurrent HRV/ENT respiratory virus may lead to a more severe clinical outcome, requiring combination therapy such as IVIG, steroids, and/or anakinra, thereby prolonging the length of hospitalization.

## Conclusions

MIS-C is an inflammatory complication of acute SARS-CoV-2 infection, and is often associated with serious end-organ involvement, warranting ICU-level of care. While MIS-C is a post-viral sequela, its co-occurrence with HRV/ENT respiratory virus may prolong hospital stay, as evidenced by our patient who required 15 days of hospitalization, which is more than twice the average of seven days reported by previous surveillance studies. Our case demonstrates the need for additional studies to better understand outcomes for patients with MIS-C and concurrent HRV/ENT infection, as well as to establish optimal treatment protocols.

## References

[1] Dionne A, Son MB, Randolph AG (2022). An update on multisystem inflammatory syndrome in children related to SARS-CoV-2. Pediatr Infect Dis J.

[2] Feldstein LR, Rose EB, Horwitz SM (2020). Multisystem inflammatory syndrome in U.S. children and adolescents. N Engl J Med.

[3] Son MB, Murray N, Friedman K (2021). Multisystem inflammatory syndrome in children - initial therapy and outcomes. N Engl J Med.

[4] Feldstein LR, Tenforde MW, Friedman KG (2021). Characteristics and outcomes of US children and adolescents with multisystem inflammatory syndrome in children (MIS-C) compared with severe acute COVID-19. JAMA.

[5] Lansbury L, Lim B, Baskaran V, Lim WS (2020). Co-infections in people with COVID-19: a systematic review and meta-analysis. J Infect.

[6] Wanga V, Gerdes ME, Shi DS (2021). Characteristics and clinical outcomes of children and adolescents aged <18 years hospitalized with COVID-19 - six hospitals, United States, July-August 2021. MMWR Morb Mortal Wkly Rep.

[7] Asner SA, Petrich A, Hamid JS, Mertz D, Richardson SE, Smieja M (2014). Clinical severity of rhinovirus/enterovirus compared to other respiratory viruses in children. Influenza Other Respir Viruses.

[8] Jiang S, Liu P, Xiong G, Yang Z, Wang M, Li Y, Yu XJ (2020). Coinfection of SARS-CoV-2 and multiple respiratory pathogens in children. Clin Chem Lab Med.

[9] Ouldali N, Toubiana J, Antona D (2021). Association of intravenous immunoglobulins plus methylprednisolone vs immunoglobulins alone with course of fever in multisystem inflammatory syndrome in children. JAMA.

